# 418. Comparing the Effects of Commercial to Manual Initial Specimen Diversion Techniques (ISDT) on Clinical Outcomes and Institutional Costs

**DOI:** 10.1093/ofid/ofae631.132

**Published:** 2025-01-29

**Authors:** Erin H Yang, Corina Lopez, Sabra Shay, Margaret Reed, Victoria McArdle, Todd M Lasco, Mayar Al Mohajer

**Affiliations:** Baylor College of Medicine, Houston, TX; Baylor College of Medicine, Houston, TX; Premier, Charlotte, North Carolina; Baylor St. Lukes Medical Center, Houston, Texas; Baylor St. Lukes Medical Center, Houston, Texas; Baylor St. Luke's Medical Center, Houston, Texas; Baylor College of Medicine, Houston, TX

## Abstract

**Background:**

Blood cultures are widely used to diagnose systemic infections. Blood culture contamination (BCC) can lead to unnecessary treatment and prolonged hospitalization. There are three primary ISDT: two commercial options (Steripath and Kurin Lock) and one manual approach (open ISDT). This study aimed to compare the effects of switching from the Steripath ISDT to open ISDT on BCC rates, length of stay (LOS), antibiotic days of therapy (DOT), and laboratory costs.

**Figure 1.**
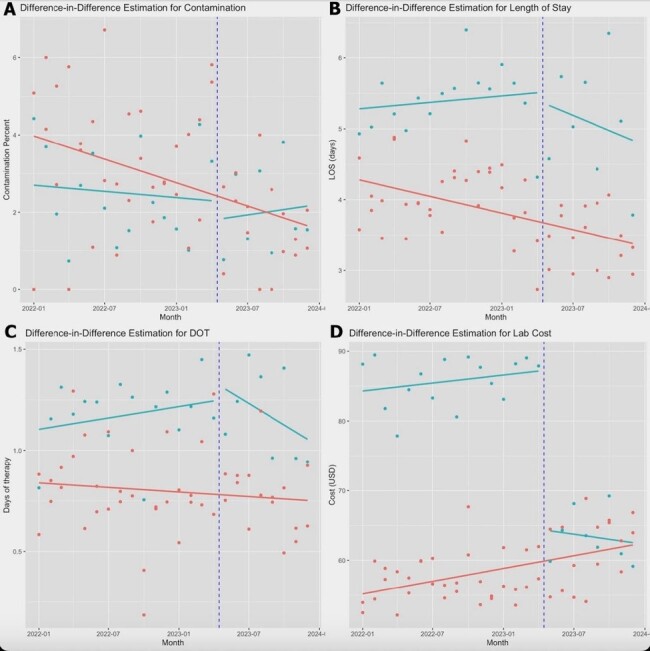
Difference-in-difference (DID) plots for Study Outcomes This figure shows difference-in-difference plots for blood culture contamination (BCC) (A), length of stay (LOS) (B), days of therapy (DOT) (C), and laboratory costs in USD (D). The green line represents the treatment group (center 1), while the red line represents the control group (centers 2 and 3). The blue dashed line represents the intervention of switching from Steripath ISDT to open ISDT.

**Methods:**

This was a non-randomized prospective controlled study conducted at three medical centers in Texas from January 2022 through December 2023. Two centers comprised the control arm, utilizing the open ISDT throughout the study period. One center served as the treatment arm where it had Steripath ISDT (pre-intervention) and then was switched to open ISDT (post-intervention). The outcomes were evaluated using multiple regression models (logistic for BCC, linear for log LOS and laboratory costs, and zero-inflated negative binomial for DOT). The difference-in-difference (DID) method compared outcome changes over time between the control and treatment groups (Figure 1).

**Table 1.**
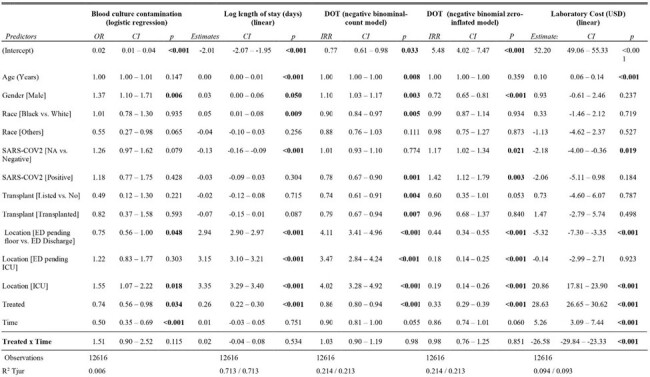
Multiple Variable Regressions for Study Outcomes This table shows the effects of switching from Steripath ISDT to open ISDT on the study outcomes: blood culture contamination (logistic regression), length of stay (logarithmic values, linear regression), days of therapy (negative binomial count and negative binomial zero-inflated models) and laboratory costs (linear regression). The Treated x Time variable represents the treatment effect. (OR = odds ratio; IRR = incidence rate ratio; DOT = days of therapy).

**Results:**

A total of 12,616 patients were included in the study, with 7,339 in the pre-intervention period and 5,277 in the post-intervention period. The intervention did not significantly impact BCC rates (OR 1.51, p=0.115, Table 1), log LOS (0.02, p=0.534), or DOT (count-model IRR 1.03, p=0.98, and zero-inflated model IRR 0.98, p=0.851). However, a significant difference in laboratory costs was observed following the switch to the open ISDT (reduction of $26.58, p< 0.001). For 1,882 patients in the treatment arm of the post-intervention period, the adjusted cost difference of $26.58 per patient implied an estimated yearly savings of $75,035.40 USD.

**Conclusion:**

Switching from Steripath to open ISDT had no significant effect on BCC rates, LOS, or antibiotic DOT. However, it did lead to a substantial reduction in laboratory costs. Considering the financial constraints faced by hospitals, this presents an opportunity for improved resource allocation. Future studies should include prospective, randomized control trials to thoroughly examine the effects of various ISDT outcomes and associated costs.

**Disclosures:**

**All Authors**: No reported disclosures

